# Supplementary material for: Cell-type-specific effects of autism-associated chromosome 15q11.2-13.1 duplications in human brain
Source: bioRxiv. 2024 May 22:2024.05.22.595175. Preprint. [Version 1] doi: 10.1101/2024.05.22.595175 (PMC11142199; doi:10.1101/2024.05.22.595175)
Supplement: Supplement 1 [file NIHPP2024.05.22.595175v1-supplement-1.pdf]

bioRxiv preprint doi: https://doi.org/10.1101/2024.05.22.595175; this version posted May 22, 2024. The copyright holder for this preprint (which was not certified by peer review) is the author/funder, who has granted bioRxiv a license to display the preprint in perpetuity. It is made available under aCC-BY 4.0 International license.

SUPPLEMENTAL FIGURES

A

	Prior Validation
**862K**	blood karyotype and FISH
**AN05983**	blood genotyping, FISH, southern blot, array CGH; brain WGS, RT-PCR of genomic DNA, RNA-seq
**AN06365**	blood genotyping, FISH, southern blot, array CGH; brain WGS, RT-PCR of genomic DNA, RNA-seq, RT-PCR
**AN11931**	blood genotyping, FISH, southern blot, array CGH; brain RT-PCR of genomic DNA, RNA-seq, RT-PCR
**AN14762**	blood genotyping, FISH, southern blot, array CGH; brain RT-PCR of genomic DNA, RNA-seq
**Z2WU**	blood karyotype and FISH

B

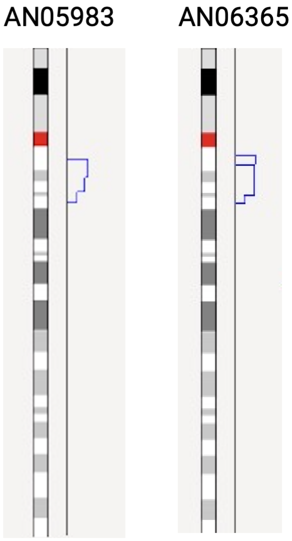

Figure S1: Dup15q sample validation (all samples have tetrasomy of the PWACR) **A.** Dup15q samples have been extensively validated in the literature[6,36]. **B.** Two samples were also validated with optical genome mapping. Note that the ideogram represents only the copy number changes and not the chromosomal structure.

bioRxiv preprint doi: https://doi.org/10.1101/2024.05.22.595175; this version posted May 22, 2024. The copyright holder for this preprint (which was not certified by peer review) is the author/funder, who has granted bioRxiv a license to display the preprint in perpetuity. It is made available under aCC-BY 4.0 International license.

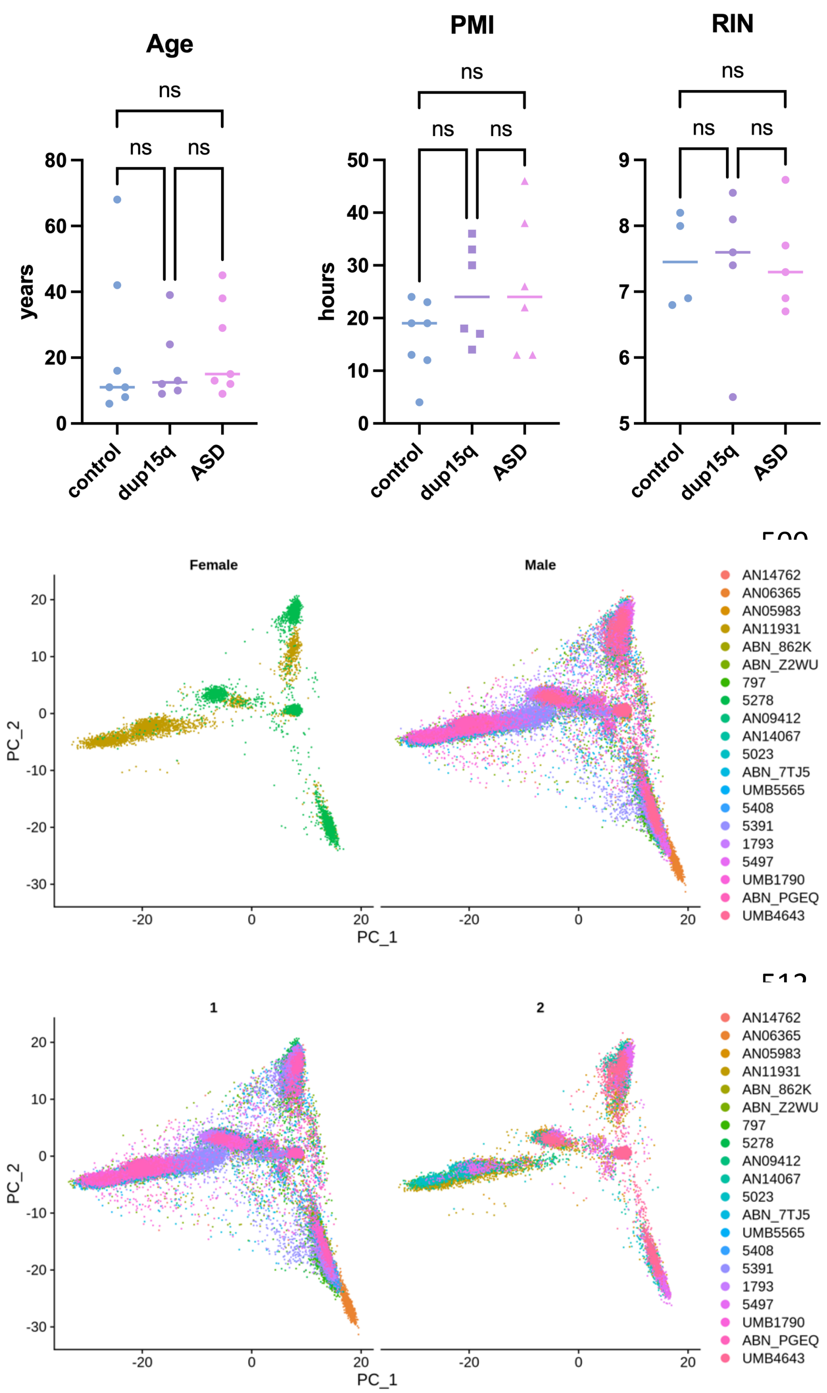

Figure S2: Top panel: Sample comparison. No significant differences in age, PMI or RIN. (p> .05, 1 way ANOVA, Tukey post-hoc test. There was also no difference in the female percentage between groups (p>.05, chi-square). Middle and bottom panel: PCA plots demonstrate sex and age (split as < and > 21 years of age) are not major determinants of gene expression variability.

bioRxiv preprint doi: https://doi.org/10.1101/2024.05.22.595175; this version posted May 22, 2024. The copyright holder for this preprint (which was not certified by peer review) is the author/funder, who has granted bioRxiv a license to display the preprint in perpetuity. It is made available under aCC-BY 4.0 International license.

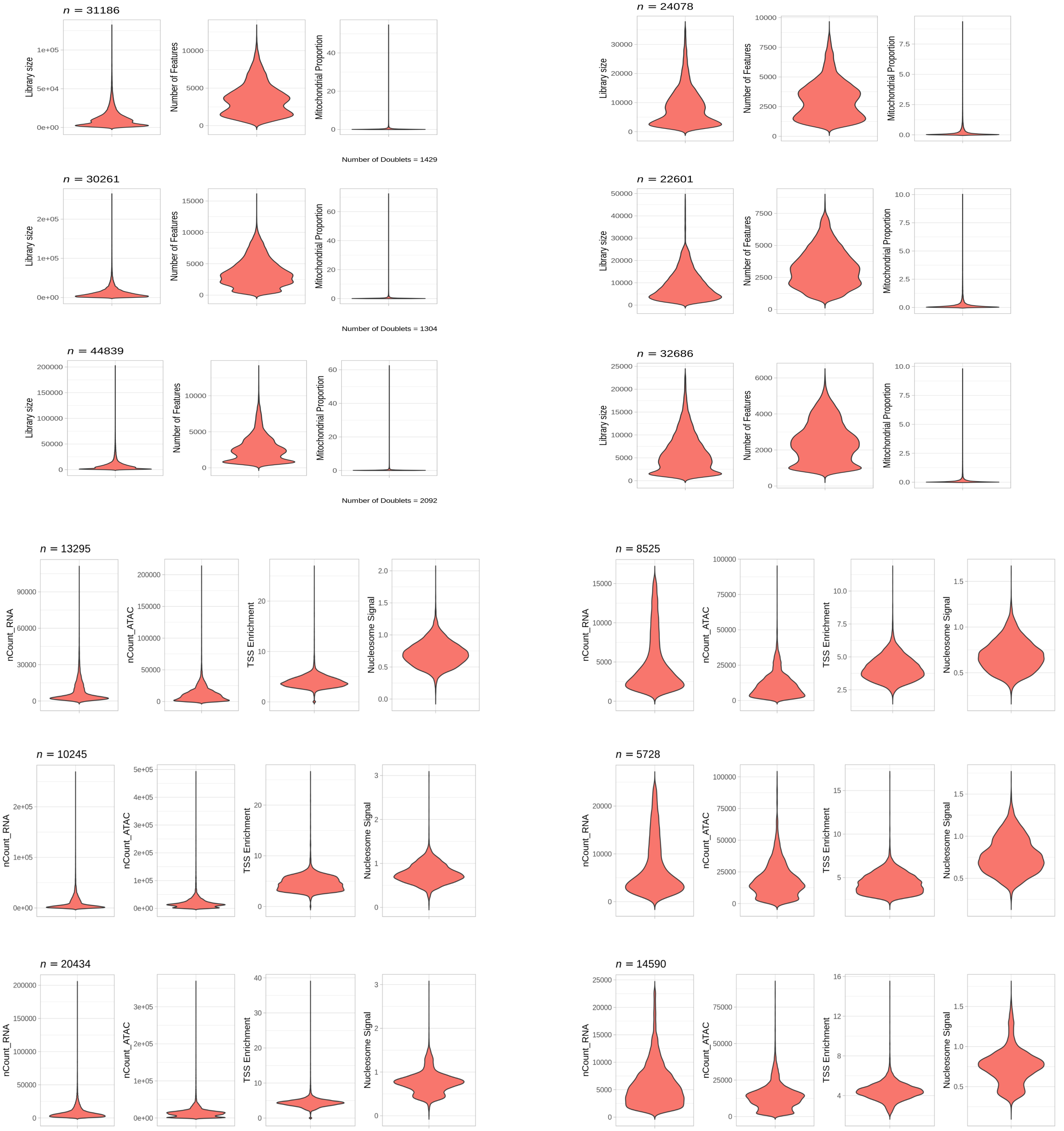

Figure S3: snRNA-seq (top 3) and multi-omic (bottom 3) quality metrics of raw (left) and filtered (right) nuclei. Each panel demonstrates library size, gene number, and proportion of mitochondrial genes (from left to right) and for ATAC-seq- ncount RNA, ATAC, TSS enrichment and nucleosome signal are shown. Top is dup15q, middle is ASD, and bottom is control. Number of nuclei in top left corner, number of doublets in each group prior to filtering also indicated.

bioRxiv preprint doi: https://doi.org/10.1101/2024.05.22.595175; this version posted May 22, 2024. The copyright holder for this preprint (which was not certified by peer review) is the author/funder, who has granted bioRxiv a license to display the preprint in perpetuity. It is made available under aCC-BY 4.0 International license.

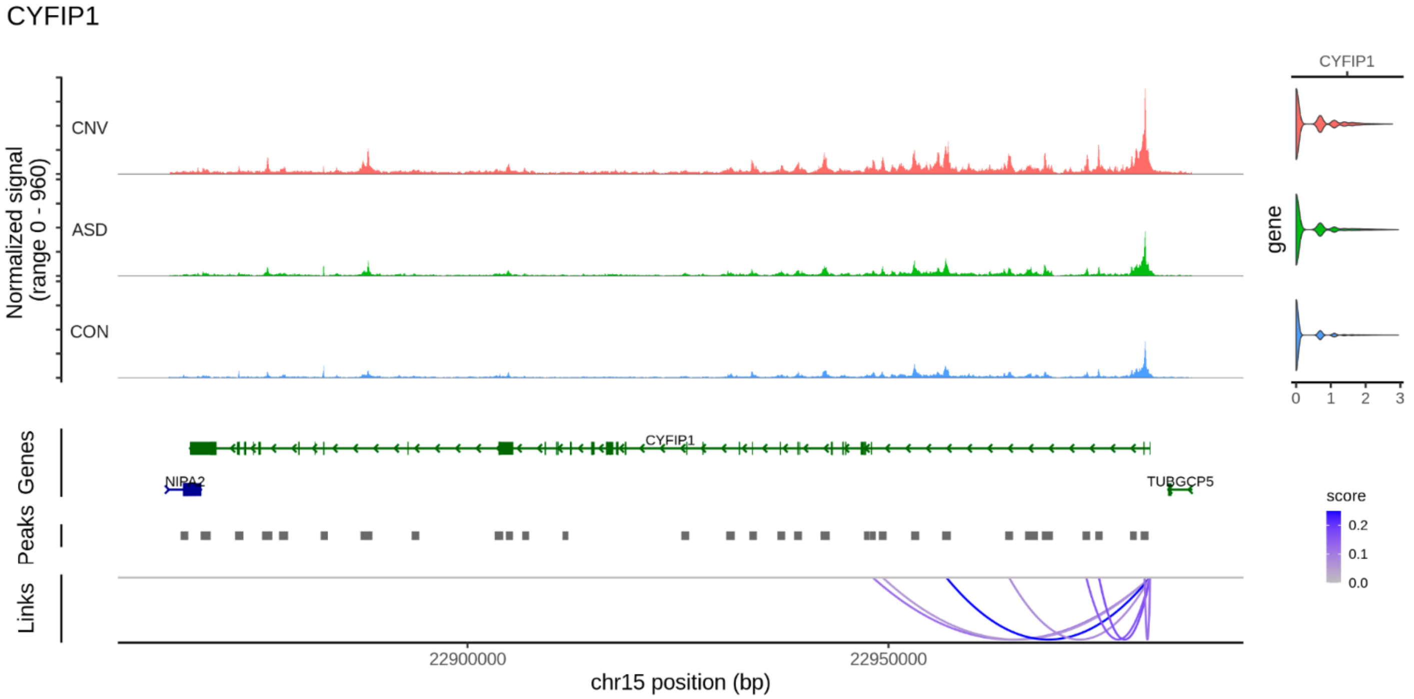

Figure S4: Increased expression and chromatin accessibility at *CYFIP1* in dup15q samples shown, as well as peak to peak linkage. Represents signal from all nuclei combined.

bioRxiv preprint doi: https://doi.org/10.1101/2024.05.22.595175; this version posted May 22, 2024. The copyright holder for this preprint (which was not certified by peer review) is the author/funder, who has granted bioRxiv a license to display the preprint in perpetuity. It is made available under aCC-BY 4.0 International license.

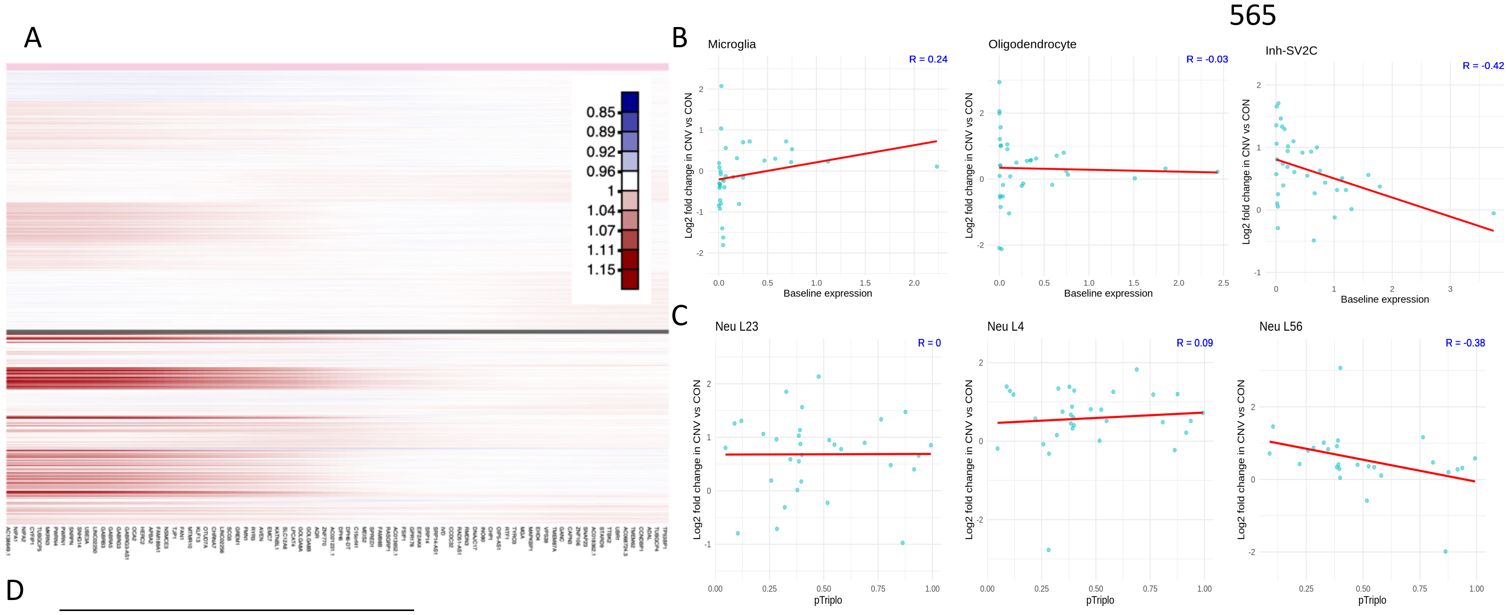

D

Gene	pTriplo
TUBGCP5	0.3846449
CYFIP1	0.87568458
NIPA1	0.52507227
NIPA2	0.39983122
MAGEL2	0.35396615
SNRPN	0.22130561
UBE3A	0.93655862
ATP10A	0.68789539
GABRB3	0.99263489
GABRA5	0.32659342
GABRG3	0.39635259
OCA2	0.08859845
HERC2	0.7635295
APBA2	0.38461863
TJP1	0.91646147
KLF13	0.47624642
OTUD7A	0.80825112
CHRNA7	0.57981061

Figure S5. Assessing mediating factors to dup15q expression changes. **A.** inferCNV reveals heterogeneous expression increases in region of duplication in dup15q cases (below grey line) across samples as compared to control (above grey line) within all nuclei (each row). Heatmap color scale indicates decreased (blue) or increased expression. **B.** Cell-type-specific examples of the association between baseline expression in controls and the fold change expression in dup15q. Within the duplicated region, genes that are highly expressed in different cell types demonstrate modest changes in expression in dup15q cases. p-values are non-significant except for in Inh-SV2C (p=.01, not adjusted for multiple comparisons) **C.** Association of pTriplo in cell-type-specific examples. All p-values > .050. **D.** pTriplo metric for select dup15q genes.

bioRxiv preprint doi: https://doi.org/10.1101/2024.05.22.595175; this version posted May 22, 2024. The copyright holder for this preprint (which was not certified by peer review) is the author/funder, who has granted bioRxiv a license to display the preprint in perpetuity. It is made available under aCC-BY 4.0 International license.

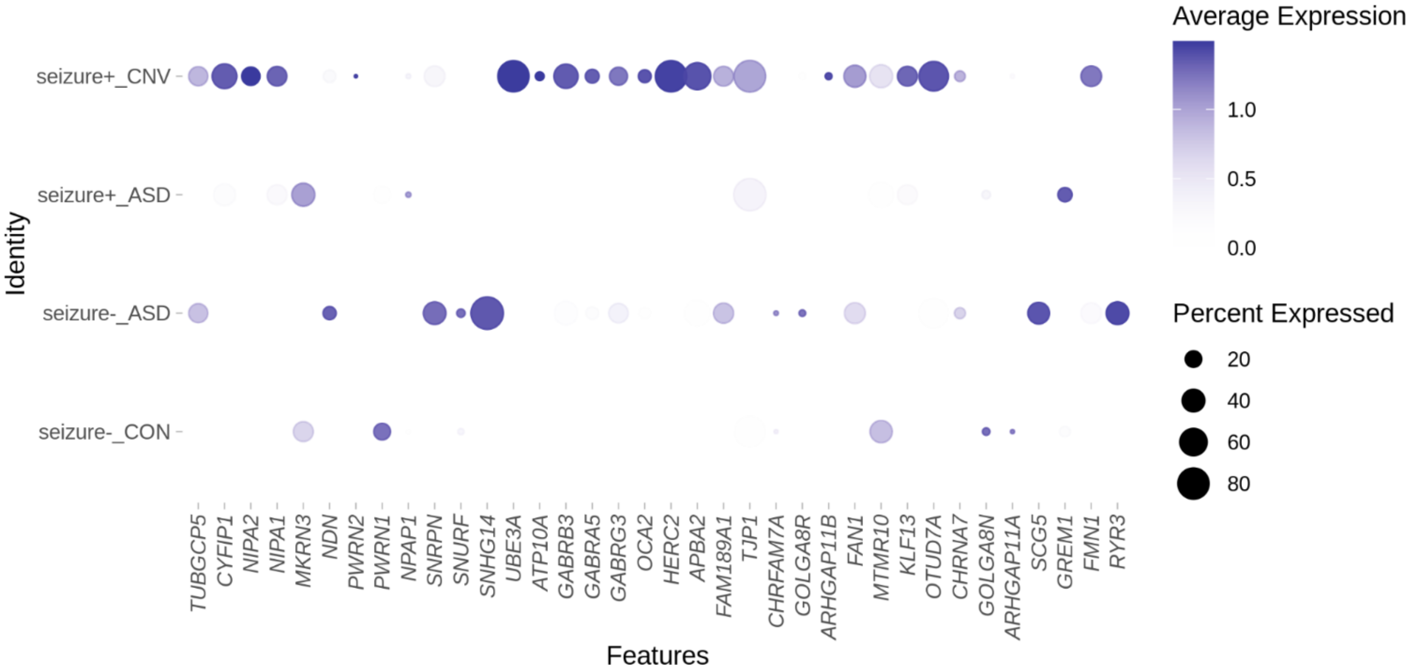

Figure S6: Presence of seizure diagnosis in ASD cases does not recapitulate dup15q gene expression changes observed within the duplicated (CNV) region.

bioRxiv preprint doi: https://doi.org/10.1101/2024.05.22.595175; this version posted May 22, 2024. The copyright holder for this preprint (which was not certified by peer review) is the author/funder, who has granted bioRxiv a license to display the preprint in perpetuity. It is made available under aCC-BY 4.0 International license.

bioRxiv preprint doi: https://doi.org/10.1101/2024.05.22.595175; this version posted May 22, 2024. The copyright holder for this preprint (which was not certified by peer review) is the author/funder, who has granted bioRxiv a license to display the preprint in perpetuity. It is made available under aCC-BY 4.0 International license.

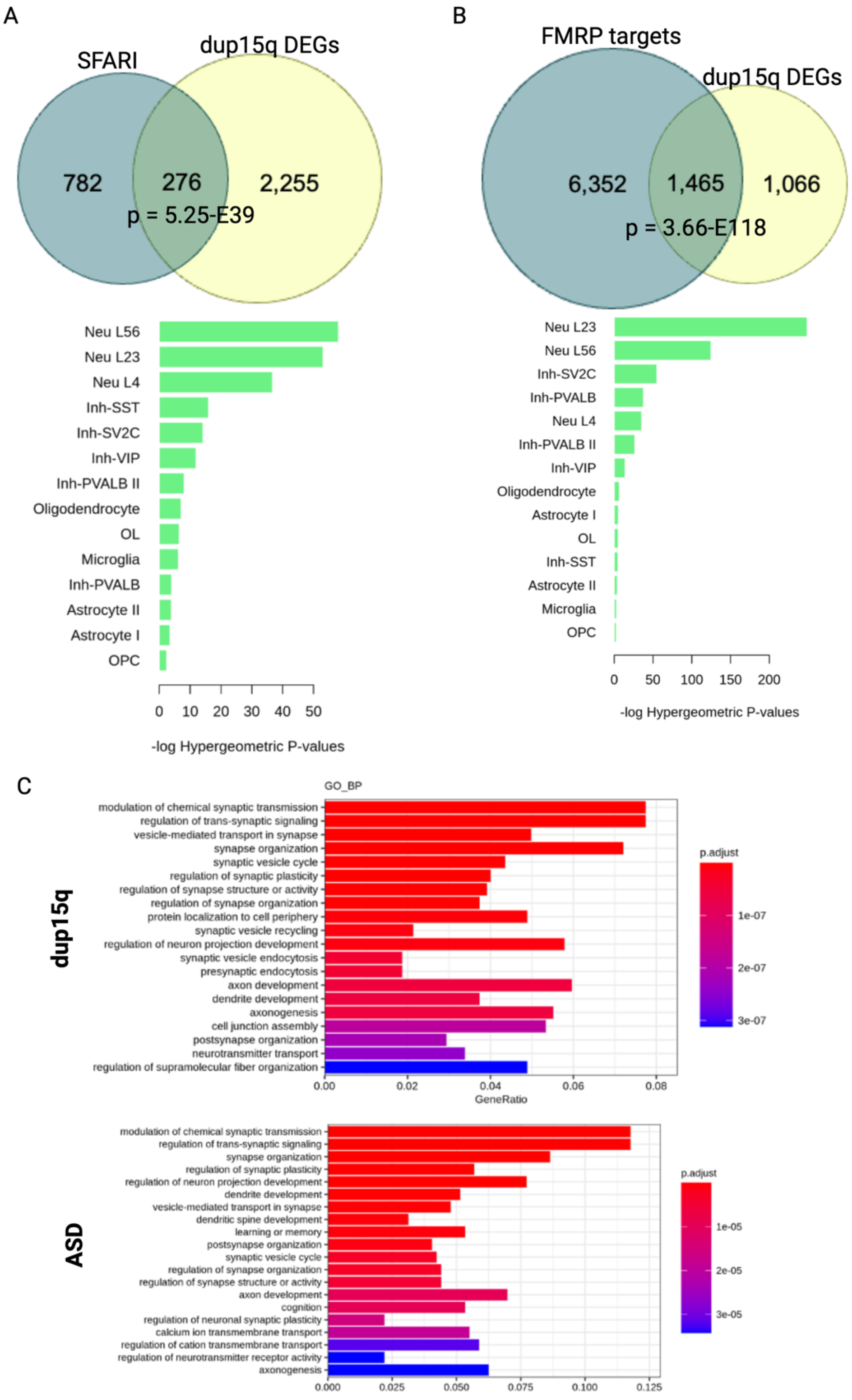

Figure S7: Overlap of known (A) SFARI pathogenic ASD risk genes as well as (B) FMR1 protein target genes, demonstrates significant enrichment (p-value indicated in figure, hypergeometric test) These genes demonstrate notable enrichment in excitatory neuron subtypes. **C.** Using gene ontology analysis, we also found evidence for biological process terms involved in synaptic function. Presented are results from differential expression analysis in layer 2/3 neurons in each condition vs. control.

bioRxiv preprint doi: https://doi.org/10.1101/2024.05.22.595175; this version posted May 22, 2024. The copyright holder for this preprint (which was not certified by peer review) is the author/funder, who has granted bioRxiv a license to display the preprint in perpetuity. It is made available under aCC-BY 4.0 International license.

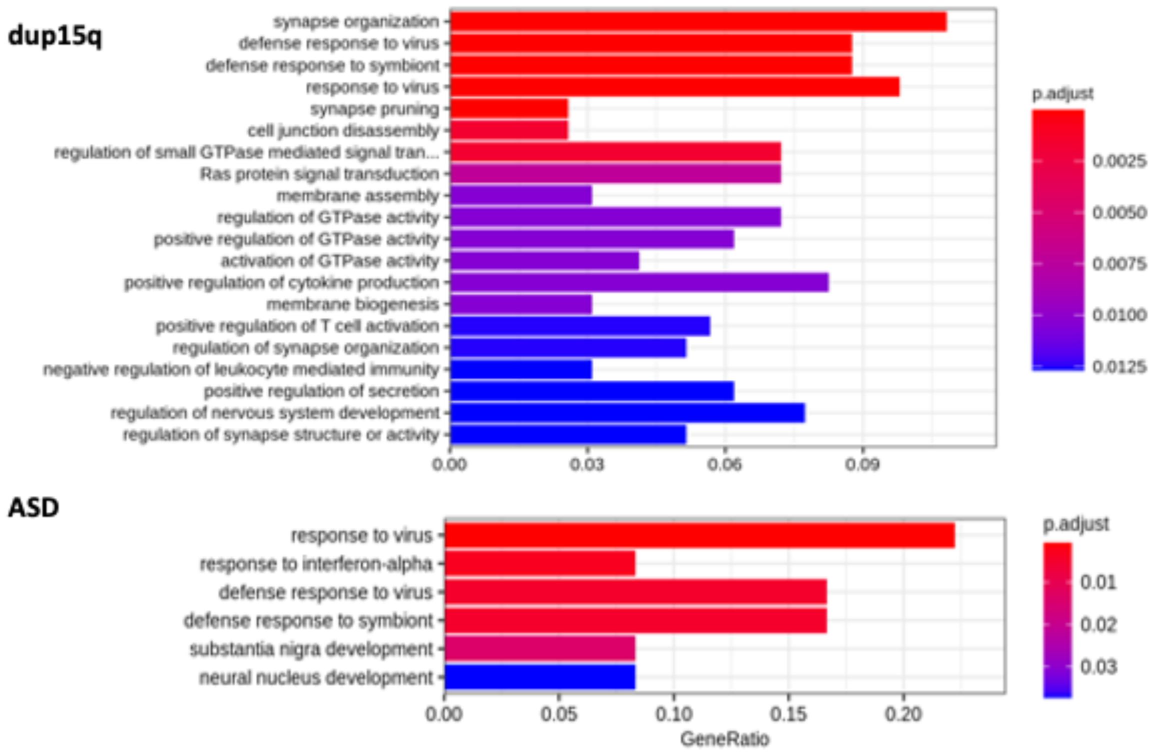

Figure S8: Using gene ontology analysis, we also found evidence for biological process terms related to inflammation and synaptic pruning in dup15q microglia, but not ASD microglia (compared to control)

bioRxiv preprint doi: https://doi.org/10.1101/2024.05.22.595175; this version posted May 22, 2024. The copyright holder for this preprint (which was not certified by peer review) is the author/funder, who has granted bioRxiv a license to display the preprint in perpetuity. It is made available under aCC-BY 4.0 International license.

All Cells	# DEGs (logfc=0.25)	# DEGs (logfc=0.2)
CNV vs CON	47	126
ASD vs CON	6	34
CNV vs ASD	23	52

Supplemental Table 1: Number of significant differentially expressed genes with different log fold change cut-off in global differential expression analysis (i.e. using all nuclei from different cell types combined).

Neuron Cells	# DEGs (logfc=0.25)	# DEGs (logfc=0.2)
CNV vs CON	259	623
ASD vs CON	111	329
CNV vs ASD	38	101

Glia Cells	# DEGs (logfc=0.25)	# DEGs (logfc=0.2)
CNV vs CON	95	206
ASD vs CON	17	45
CNV vs ASD	35	113

Supplemental Table 2: Number of significant differentially expressed genes with different log fold change cut-off in differential expression analysis separating neuron and glia.

bioRxiv preprint doi: https://doi.org/10.1101/2024.05.22.595175; this version posted May 22, 2024. The copyright holder for this preprint (which was not certified by peer review) is the author/funder, who has granted bioRxiv a license to display the preprint in perpetuity. It is made available under aCC-BY 4.0 International license.

Cell Type	# DEGs	# Upreg	# Downreg	Cell Type	# DEGs	# Upreg	# Downreg
Non-neuronal				Neuronal			
Pericyte	48	23	25	Neu L56	3	2	1
Endothelial	11	7	4	Neu L4	308	239	69
Microglia	240	124	116	Neu L23	1,307	1,162	145
Astrocyte I	115	60	55	Inh-SV2C	315	290	25
Astrocyte II	124	82	42	Inh-VIP	107	84	23
Oligodendrocyte	119	78	41	Inh-PVALB	175	165	10
OL	5	3	2	Inh-SST	74	40	34
OPC	22	14	8	Inh-PVALB II	205	193	12
T Cells	1	1	0				

Supplemental Table 3: Number of significant differentially expressed genes in high resolution differential expression analysis, in dup15q vs control comparison (p-value adj<0.05).
